# Human peripheral blood mononuclear cells display a temporal evolving inflammatory profile after myocardial infarction and modify myocardial fibroblasts phenotype

**DOI:** 10.1038/s41598-023-44036-3

**Published:** 2023-10-05

**Authors:** Elodie Miquelestorena-Standley, Ana Valéria Vinhais da Silva, Marina Monnier, Stéphanie Chadet, Marie Piollet, Audrey Héraud, Roxane Lemoine, Thomas Bochaton, Geneviève Derumeaux, Sébastien Roger, Fabrice Ivanes, Denis Angoulvant

**Affiliations:** 1 EA 4245 Transplantation, Immunologie, Inflammation, Faculté de Médecine, Université de Tours, 10 boulevard tonnele, 37032 Tours Cedex 1, France; 2 Service d’Anatomie et Cytologie Pathologiques, CHRU de Tours, Tours, France; 3 Service de Cardiologie, Hospices Civils de Lyon, Lyon, France; 4 Service de Physiologie, Hôpital Henri Mondor, AP-HP, Université Paris-Est Créteil, INSERM U955, Créteil, France; 5 Service de Cardiologie, CHRU de Tours, Tours, France

**Keywords:** Cardiology, Medical research

## Abstract

Pathophysiological response after acute myocardial infarction (AMI) is described as a three-stage model involving temporal phenotypic modifications of both immune cells and fibroblasts: a primary inflammatory phase, followed by a reparative phase and a fibrous scar maturation phase. Purinergic receptors, particularly the P2Y11 receptor, have been reported to be involved in the regulation of inflammation after ischemia and could act for the resolution of inflammation after AMI. For the first time, we characterized the immuno-inflammatory and P2Y11 expression profiles of peripheral blood mononuclear cells (PBMC) from AMI patients and analyzed the consequences of presenting these cells to cardiac fibroblasts in vitro. PBMC from 178 patients were collected at various times after reperfused ST-segment elevation AMI, from H0 to M12. Expression level of *P2RY11* and genes involved in tolerogenic profile of dendritic cells and T cell polarization were evaluated by RT-PCR. P2Y11 protein expression was assessed by flow cytometry. PBMC and human cardiac fibroblasts (HCF) were cocultured and α-SMA/vimentin ratio was analyzed by flow cytometry. Within the first 48 h after AMI, expression levels of *HMOX1*, *STAT3* and *CD4* increased while *IDO1* and *TBX21/GATA3* ratio decreased. Concomitantly, the expression of *P2RY11* increased in both T and B cells. In vitro, PBMC collected at H48 after AMI induced an increase in α-SMA/vimentin ratio in HCF. Our results suggest that human PBMC display an evolving inflammatory profile with reparative characteristics the first two days after AMI and secrete soluble mediators leading to the fibroblastic proteins modification, thus participating to myocardial fibrosis.

## Introduction

Cardiovascular diseases represent leading causes of death in the world with about 1.7 million deaths per year. Among these, coronary heart disease is the first cause of cardiovascular deaths in the United States (42.1%)^1^.

Within the last decades, the pathophysiology of myocardial infarction has been well explored and described in several murine models. The current description of wound healing leading to myocardial fibrosis is based on a three-stages model reporting a temporal evolution of immune cells infiltrating the myocardium and modifications of fibroblasts phenotype^2–6^. Acute coronary artery occlusion is often responsible for oxygen and nutrients deprivation leading to myocardial cells necrosis and release of alarmins. This phenomenon leads to sterile inflammation and recruitment of neutrophils to the infarct site. During the first “inflammatory phase”, occurring between 1 and day 4 in mice, pro-inflammatory cells such as M1 macrophages, Th1 and B cells are recruited and activated in the infarct site and a proliferation of cardiac fibroblasts is observed. During the “reparative phase”, from 3 to day 14, resolutive M2 macrophages and T regulatory cells regulate inflammation and modulate fibroblasts profile. Fibroblasts acquire a myofibroblast phenotype with contractile abilities due to an increase of α-smooth muscle actin and a reduction of vimentin, as well as an increase of collagen secretory properties participating to the development of the extracellular matrix. Finally, during the “maturation phase”, the fibrous scar becomes more complex and stable, and activated fibroblasts transform into matrifibrocytes that lose α-smooth muscle actin expression and proliferative capacities^6^.

While the lack of efficient wound healing driven by inflammation can lead to the rupture of ventricular myocardium, an excessive or extended immune response can be responsible for adverse remodeling with pejorative clinical consequences. In fact, ventricular remodeling involves interactions between both immune cells and cardiac fibroblasts through various complex and finely regulated pathways^7–10^. Several studies reported the crucial role of macrophages, monocytes or T cells, and of various mediators like cytokines and lipid mediators, in inflammation and healing after acute myocardial infarction (AMI)^3,4,8,9,11–16^. Among other mediators studied, extracellular ATP (eATP) released by dying and stressed cells may be involved in this active and complex mechanism by acting on purinergic receptors which roles in the pathogenesis of myocardial damages represent a promising research topic for therapeutic management^17^. We previously observed, in in vitro and in vivo murine models that the P2Y11 receptor has an important role in the immune-inflammatory response to ischemia/reperfusion. This ubiquitous receptor coupled with both Gq and Gs α-subunits is activated by ATP, leading to activation of phosphoinositide phospholipase C and adenylyl cyclase pathways respectively^18,19^. We and others observed that its activation has anti-inflammatory and anti-fibrotic effects, and that it is involved in T cells migration and M2 macrophages differentiation^18,20–24^. We first revealed that this receptor plays a role in eATP-induced dendritic cells maturation and that hypoxia/reoxygenation induces a reduction of P2Y11 expression through a HIF-1α-dependent mechanism, thus sustaining a deleterious inflammatory response^21^. We also reported that the activation of P2Y11 reduces myocardial ischemia/reperfusion injuries and improved cardiac graft survival in a mouse model of allogeneic heart transplantation^20^. These properties are of considerable interest for the understanding of AMI pathophysiology as for the prevention of adverse cardiac remodeling, however its expression and role have not yet been studied in patients.

We hypothesize that ischemia/reperfusion lesions following reperfused myocardial infarction could induce variations in inflammatory, immune and P2Y11 expression profile in peripheral cells of patients, and that these cells could participate in cardiac remodeling through modulation of cardiac fibroblasts phenotype.

The aims of our study were 1/to study the peripheral immuno-inflammatory response of patients after myocardial infarction, 2/to evaluate the expression of P2Y11 in peripheral immune cells of patients after AMI, 3/to study the in vitro effects of peripheral immune cells of patients on cardiac fibroblasts activation.

## Results

### Clinical data

Characteristics of AMI patients are summarized in Table 1. We collected 415 samples from 178 ST elevation myocardial infarction (STEMI) patients (26 patients from the Hibiscus-STEMI cohort and 152 from the CARIM cohort) and 30 healthy donors. Eighty percent (80%) of patients were male and median age was 60 years. Forty-one percent (41%) were active smokers, 5% had diabetes, 36% had dyslipidemia, and 38% had hypertension at the time of AMI. The observed median of duration of ischemia was 184 min. Fifty-two percent (52%) had single, 29% had two-, and 19% had three-vessel disease on coronary angiography. Fourty-nine percent of patients had a 0 TIMI flow score before reperfusion. Hypersensitive Troponin T median concentration was 121.3 µg/L (N < 14 µg/L) at the time of admission and peak median concentration was 2582 µg/L. One year after inclusion the median left ventricular ejection fraction was 55%.

**Table 1:** Clinical and biological characteristics of cohorts.

	n = 178
Male, Female (n(%))	142 (80%), 36 (20%)
Age, years (median (interquartile range))	60 (55–68)
Active smoker (n(%))	71 (41%)
Body Mass Index, kg/m^2^ (median (interquartile range))	26.7 (24.2–29.4)
Family history of cardiovascular disease (n(%))	51 (29%)
Diabetes mellitus (n(%))	9 (5%)
Dyslipidemia (n(%))	61 (36%)
Hypertension (n(%))	66 (38%)
Ischemia duration, minutes (median (interquartile range))	184 (145–259)
Number of coronary artery stenosis: 0, 1, 2, 3	0 (0%), 92 (52%), 51 (29%), 33 (19%)
TIMI flow score prior to reperfusion: 0, 1, 2, 3	86 (49%), 6 (3%), 15 (9%), 68 (39%)
H0 Hypersensitive Troponin T, µg/L (median (interquartile range))	121.3 (29.6–494.3)
Hypersensitive Troponin T peak, µg/L (median (interquartile range))	2,582 (1261–4422)
M12 Left ventricular ejection fraction, % (median (interquartile range))	55 (50–59)

### A tolerogenic and anti-inflammatory profile is observed in plasma and PBMC at the early phase post-AMI

#### Chemokines and cytokines vary in plasma the first 3 days after reperfused AMI

**Figure 1:**
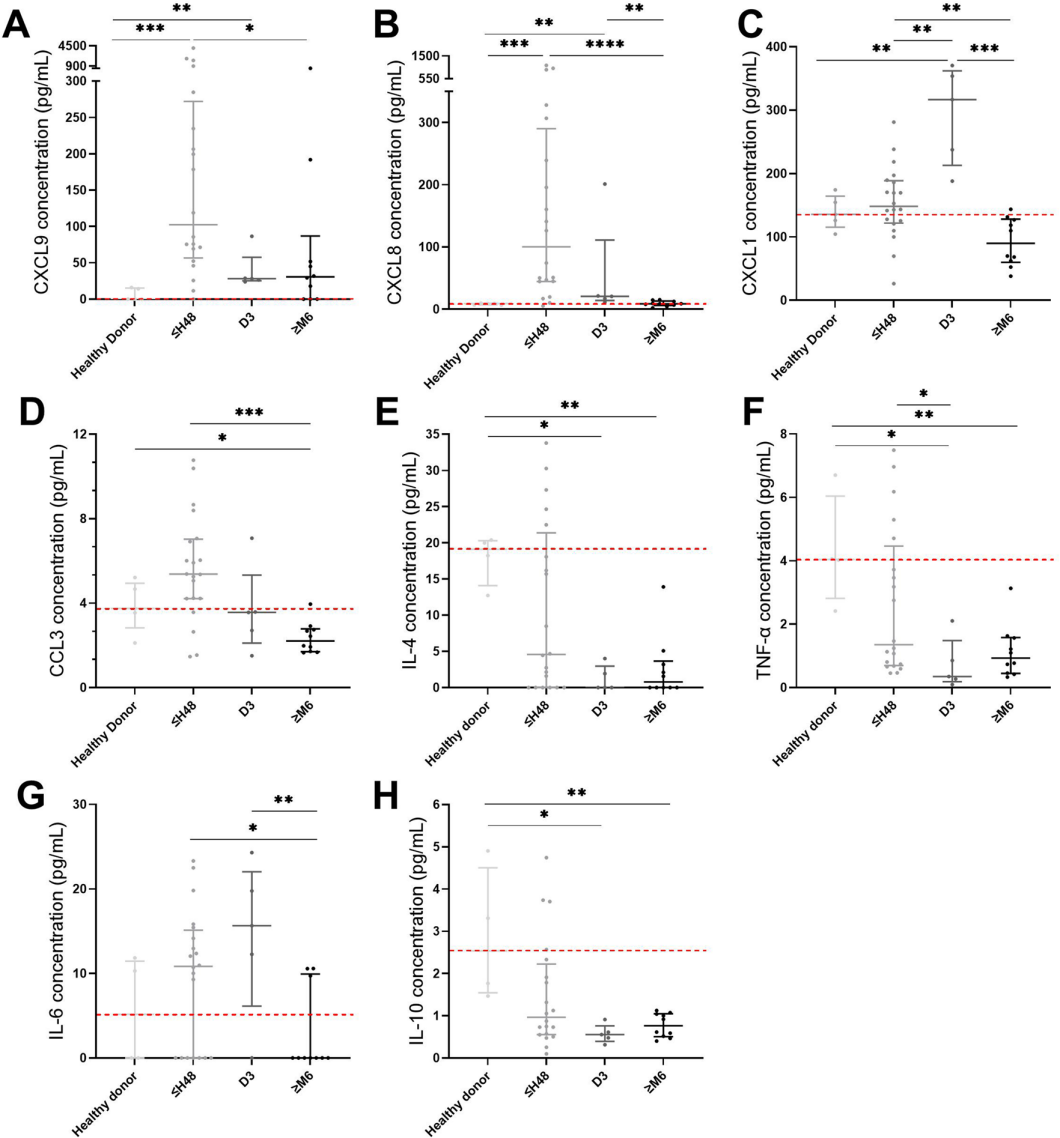
Chemokines (**A**–**B**) and cytokines (**E** and **H**) concentration (pg/mL) in plasma of healthy donors and patients after myocardial infarction. (**A**) CXCL9 (MIG), (**B**) CXCL8 (IL-8), (**C**) CXCL1 (GRO-α), (**D**) CCL3 (MIP-1α), (**E**) IL-4, (**F**) TNF-α, (**G**) IL-6, (**H**) IL-10. n = 4 samples in healthy donors, n = 20 samples in ≤ H48 group, n = 5 samples in D3 group, n = 10 samples in ≥ M6 group. Error bars correspond to interquartile range. Kruskall-Wallis test was used to compare the 4 groups and the Mann–Whitney rank sum test to compare 2 groups. *p < 0.05, **p < 0.01, ***p < 0.005, ****p < 0.001.

We analyzed concentrations of different chemokines in plasma collected from healthy donors (n = 4) and patients at various time after AMI (n = 5 samples for each time) (Fig. 1) (for detailed time-points concentrations see Supplementary Fig. S1 online). CXCL8 (IL-8) level was higher in patients between H0 and H48 (100.3 pg/mL for H0–H48 group vs. 9.19 pg/mL for healthy donors, *p* < 0.0001), CXCL1 (GRO-α) was higher in patients at D3 (316.8 pg/mL at D3 vs. 135.7 pg/mL for healthy donors, *p* = 0.0005) and CXCL9 (MIG) was higher in patients between H0 and H48 (102.3 pg/mL for H0–H48 group vs. 0 pg/mL for healthy donors, *p* = 0.0016) as compared to healthy donors. Furthermore, CCL3 (MIP-1α) showed higher levels in patients between H0 and H48 as compared to M6-M12 (5.375 at H0–H48 vs. 2.21 pg/mL respectively, *p* = 0.0032). Other chemokines were analyzed (CXCL-10, CCL-17, CCL-2, CCL-5, CXCL-11, CCL-4) but did not show any significant variation (data not shown).

We then analyzed cytokine concentrations in the same plasma samples. IL-4, IL-10 and TNF-α concentrations were lower in patients from D3 to M12 as compared to healthy donors whereas IL-6 concentration was higher in patients at early times as compared to M6-M12 samples (see Supplementary Fig. S2 online).

These data suggested that there was an acute systemic response related to the recruitment of neutrophils (CXCL-8, CXCL-1) and lymphocytes (CXCL-9, CCL-3) in the infarct site, and a polarization of T cells (CXCL-9, IL-6, IL-4), that could be recorded as early as 48 h after reperfused AMI.

#### PBMC collected early after AMI display a decreased capacity to secrete cytokines

**Figure 2:**
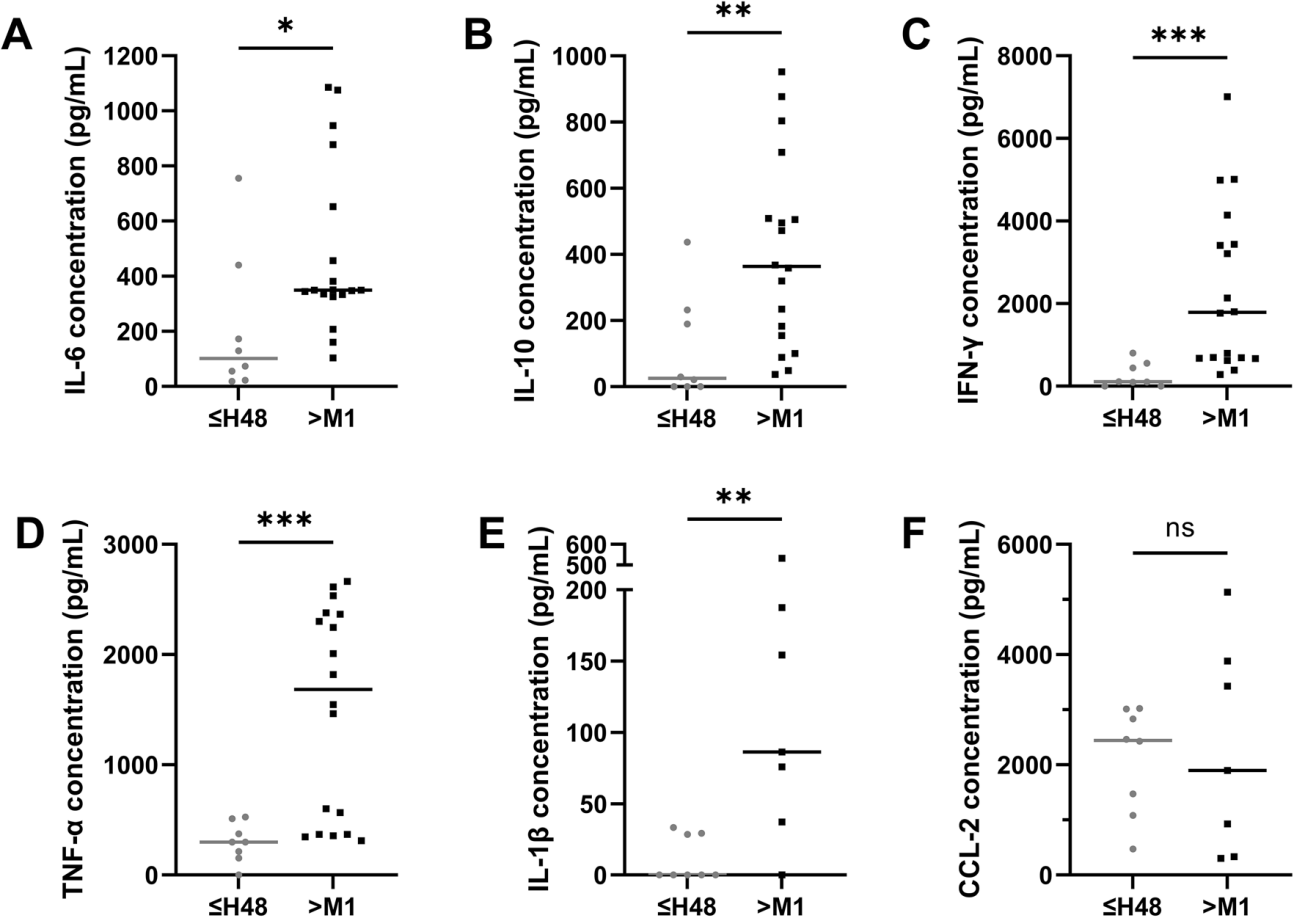
Cytokines and chemokine concentrations (pg/mL) in supernatants of PBMC stimulated with PMA/ionomycin. (**A**) IL-6, (**B**) IL-10, (**C**) IFN-γ, (**D**) TNF-α, (**E**) IL-1β, (**F**) CCL-2. n = 8 samples in ≤ H48 group, n = 18 samples in > M1 group for IL-6, IL-10, IFN-γ, TNF-α and n = 7 samples for IL-1β and CCL-2. Error bars correspond to interquartile range. Mann–Whitney rank sum test was used to compare the 2 groups. *p < 0.05, **p < 0.01, ***p < 0.005. ns stands for no statistical difference.

PBMC were collected within the first 48 h after AMI (8 samples) and their capacities to secrete cytokines and chemokines after overnight stimulation with PMA/ionomycin were compared to cells collected from 1 to 12 months after AMI (7 to 18 samples) (Fig. 2). Supernatants of PBMC collected within the first 48 h after AMI contained lower concentrations of pro-inflammatory (IL-6: *p* = 0.00158, IL-1β: *p* = 0.0041, TNF-α: *p* = 0.0008, IFN-γ: *p* = 0.0003) or anti-inflammatory (IL-10: *p* = 0.0041) cytokines compared to that of PBMC collected after the first month after AMI. Only the capacity to secrete CCL-2 chemokine was similar in both groups.

These results suggested that PBMC collected within the first 48 h after AMI displayed a decreased ability to secrete both pro- and anti-inflammatory cytokines but a preserved chemokine secretion as compared to PBMC collected after the first month post-AMI.

Since concentrations of both cytokines and chemokines in plasma and PBMC secretion abilities varied according to time after AMI (with the most important variations occurring within the first 48 h), we then studied the expression of genes and proteins, involved in dendritic cells tolerogenic profile and T cells polarization.

#### PBMC adopt a transcriptional reparative profile within the first two days after AMI

**Figure 3:**
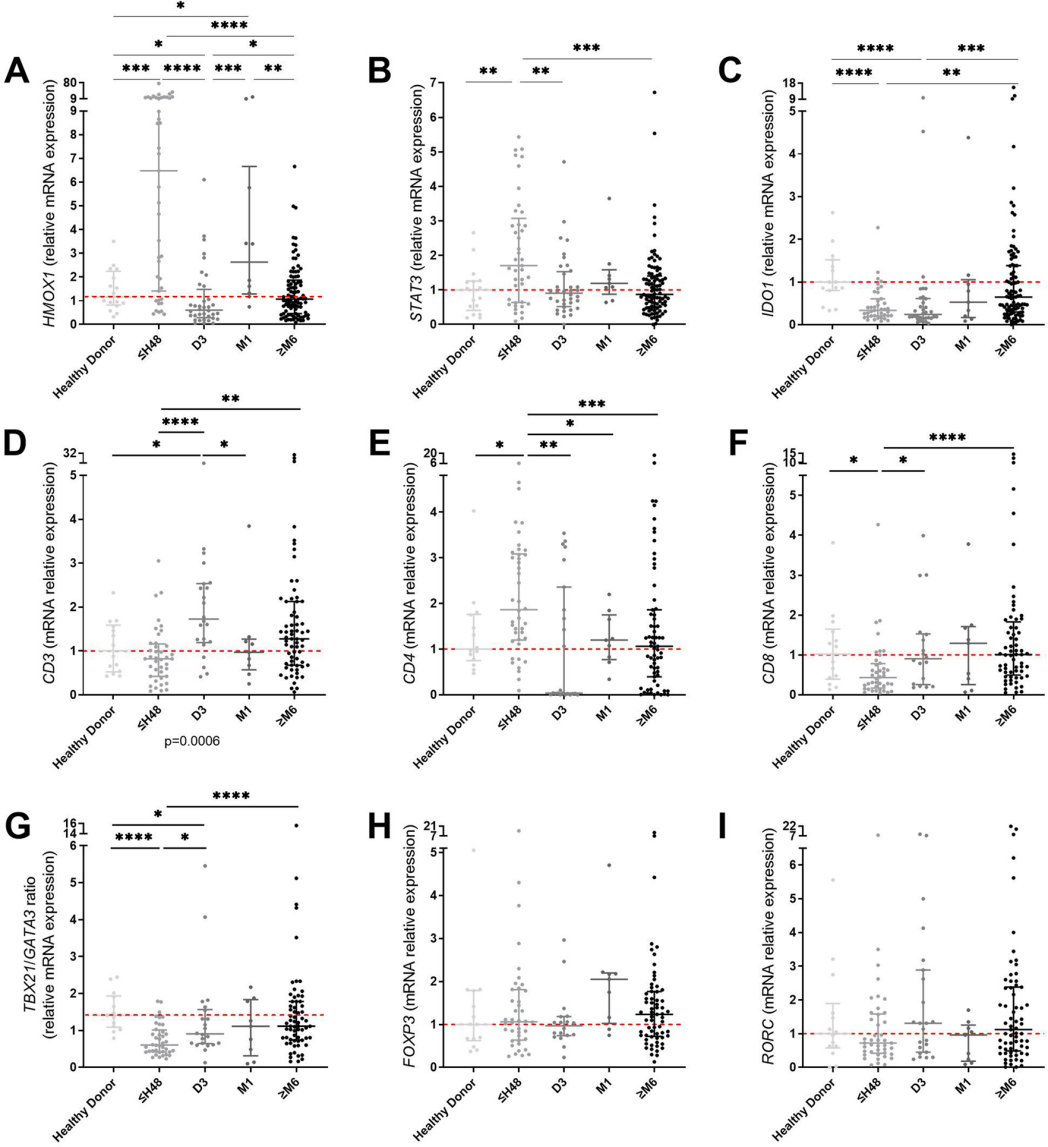
Expression levels of genes involved in tolerogenicity profile of dendritic cells (**A**–**C**) and of T cells genes (**D**–**I**). (**A**) *HMOX1*, (**B**) *STAT3*, (**C**) *IDO1*, (**D**) *CD3*, (**E**) *CD4*, (**F**) *CD8*, (**G**) *TBX21*/*GATA3* ratio, (**H**) *FOXP3*, (**I**) *RORC*. n = 14 to 17 samples in healthy donors, n = 40 to 43 samples in ≤ H48 group, n = 18 to 37 samples in D3 group, n = 9 to 10 samples in M1 group, n = 64 to 95 samples in ≥ M6 group. Error bars correspond to interquartile range. Kruskall-Wallis test was used to compare the 4 groups, Mann–Whitney rank sum test to compared 2 groups. *p < 0.05, **p < 0.01, ***p < 0.005, ****p < 0.0001.

The expression of *HMOX1, IDO1* and *STAT3*, genes known to regulate dendritic cells tolerogenicity^25^ was studied in all collected samples (Fig. 3) (for detailed time-points levels of expression see Supplementary Fig. S3 online). *HMOX1* and *STAT3* demonstrated a significantly higher level of expression during the first 48 h after AMI as compared to healthy donors (fold-change = 6.48 for H0-H48 group vs. 1.16 for healthy donors, *p* < 0.0001 and 1.71 for H0-H48 group vs. 1 for healthy donors, *p* = 0.0031 respectively) and then returned to baseline at M6. *IDO1* decreased in PBMC from all patients, from H0 to M12, compared to healthy donors (fold-change = 0.24 at D3 vs. 0.99 for healthy donors, *p* < 0.0001).

The expression of genes associated with T cell polarization was also analyzed by RT-qPCR in PBMC. Between H0 and H48, *CD4* levels were higher in patients (fold-change = 1.86 for H0-H48 group vs. 1 for healthy donors, *p* = 0.0018), while *CD8* levels (fold-change = 0.43 for H0-H48 group vs. 1.02 for healthy donors, *p* = 0.0023), and *TBX21*/*GATA3* ratio, corresponding to Th1/Th2 ratio, were lower (fold-change = 0.6 for H0-H48 group vs. 1.41 for healthy donors, *p* = 0.0001). At M1 and M6-M12, expression levels of these genes showed no difference with the healthy donor group. *CD3* was higher in patients at D3 (fold-change = 1.73 at D3 vs. 1.01 for healthy donors, *p* = 0.0005), but displayed no difference with the healthy donor group at M1 and M6–M12. We did not observe any significant variation of both *FOXP3* (fold-change = 1.06 for H0-H48 group and 2.05 at M1 vs. 1 for healthy donors, *p* = 0.1656) and *RORC* (fold-change = 0.72 for H0–H48 group vs. 1 for healthy donors, *p* = 0.2807) expression in patients compared to healthy donors (Fig. 3).

These data suggested that PBMC displayed a pro-tolerogenic profile and Th1/Th2 imbalance in favor of the Th2 phenotype at transcriptional level within the first 48 h after AMI.

**Figure 4:**
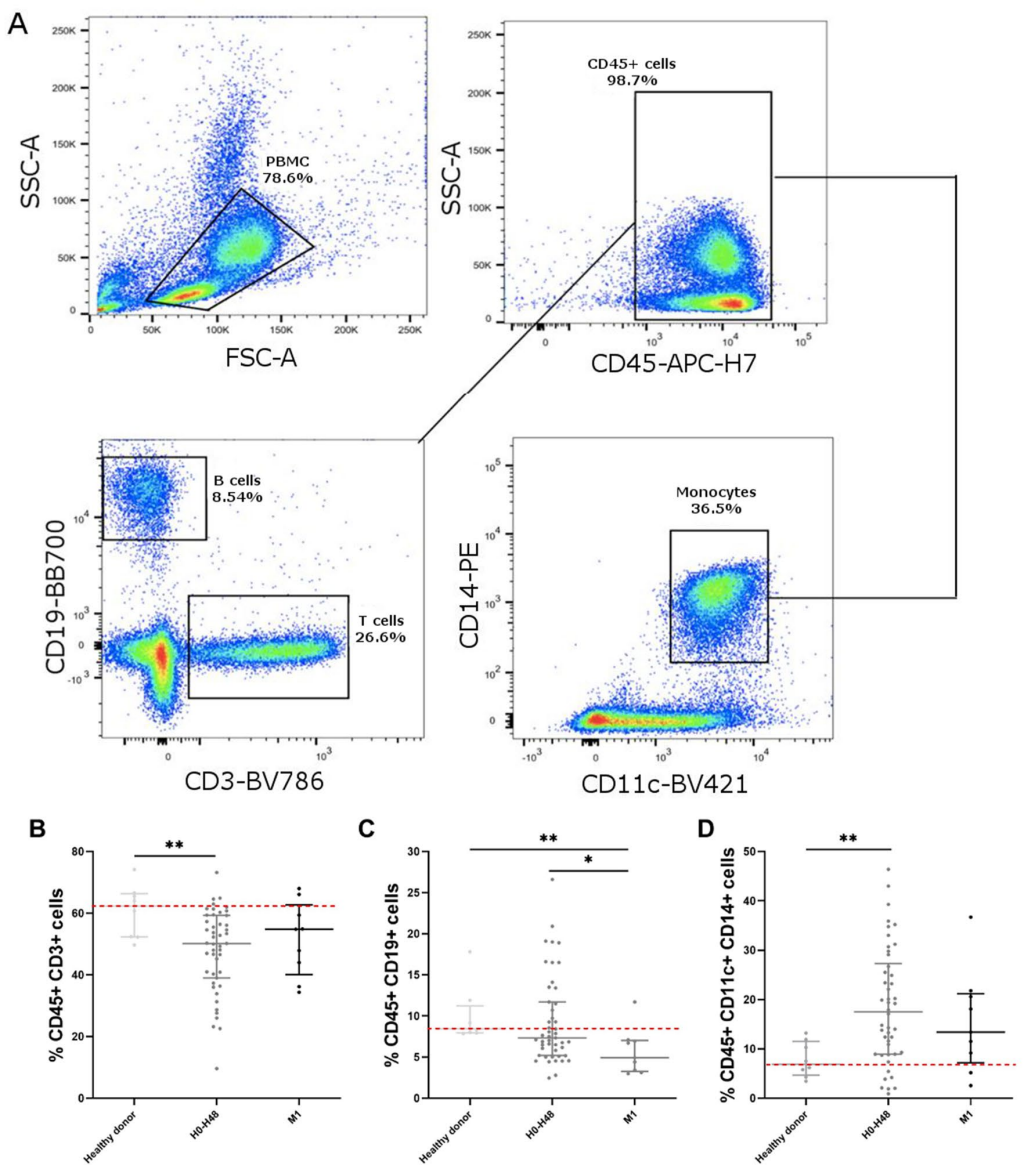
Subpopulations of PBMC by flow cytometry. (**A**) gating strategy of PBMC, CD45 + CD19 + , CD3 + and CD14 + CD11c + cells, (**B**) CD3 + (% of CD45 + cells), (**C**) CD19 + (% of CD45 + cells), (**D**) CD14 + CD11c + (% of CD45 + cells). n = 8 samples in healthy donors, n = 47 samples in H0-H48 group, n = 9 samples in M1 group. Error bars correspond to interquartile range. Kruskall-Wallis test was used to compare the 4 groups, Mann–Whitney rank sum test to compared 2 groups. *p < 0.05, **p < 0.01.

We performed flow cytometry in PBMC of 16 STEMI patients from the Hibiscus-STEMI cohort and 8 healthy donors (Fig. 4). We observed a decrease in T CD3 + cells within the first 48 h after AMI (50.2% for H0–H48 vs. 62.4% for healthy donors, *p* = 0.0375) and of B CD19 + cells within the first month after AMI (4.9% at M1 vs. 8.4 for healthy donors, *p* = 0.0161). The percentage of monocytes CD11c + CD14 + increased the first 48 h after AMI (17.5% for H0–H48 group vs. 6.9 for healthy donors, *p* = 0.0311).

### The overexpression of P2Y11 receptor in PBMC during the first two days after AMI suggests its role in the anti-inflammatory profile

**Figure 5:**
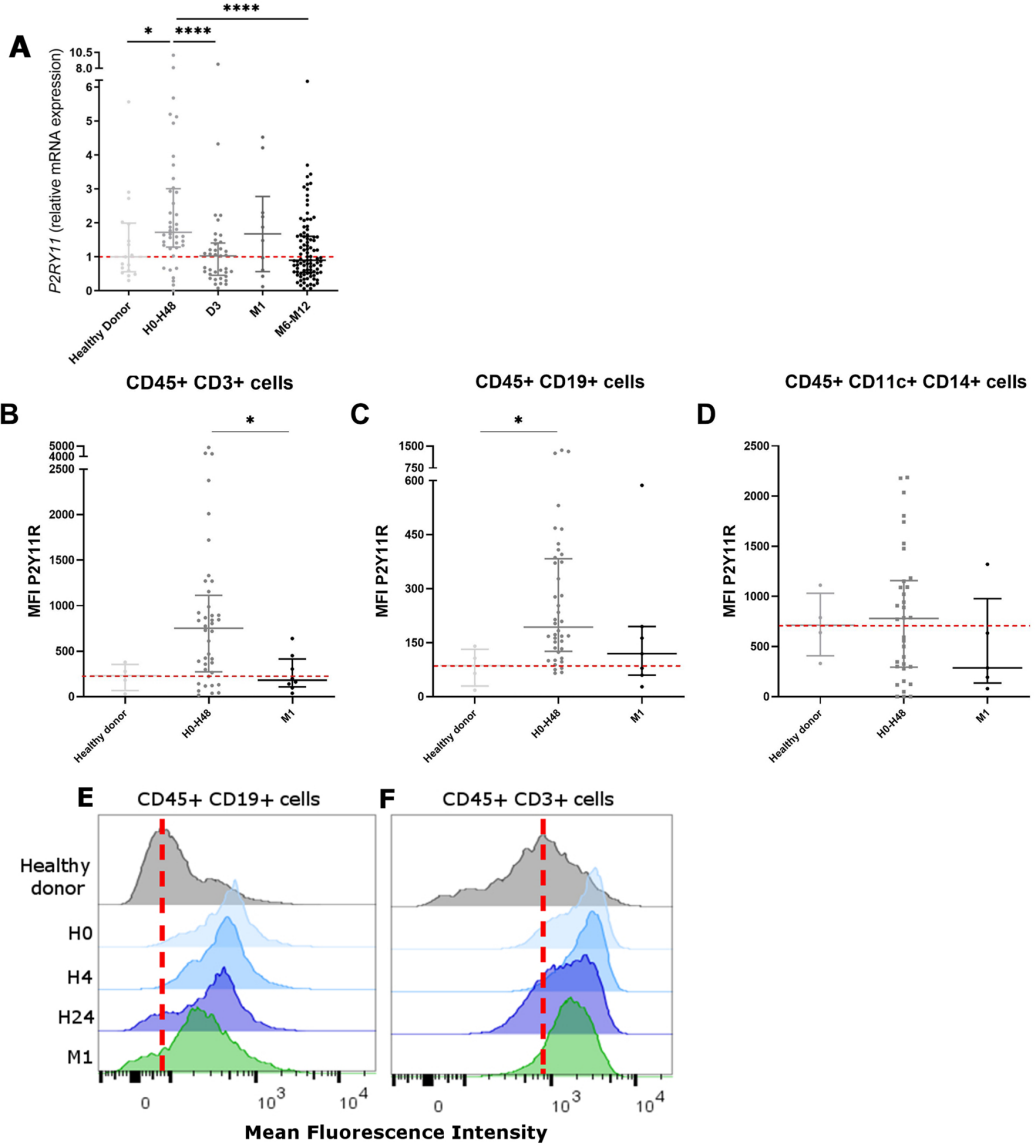
Expression level of the *P2RY11* gene was analyzed by RT-qPCR (**A**) and P2Y11 protein expression by flow cytometry (MFI) in CD3 + cells (**B**,**E**), CD19 + cells (**C**,**f**) and CD11c + CD14 + cells (**D**). RT-qPCR: n = 17 samples in healthy donors, n = 40 samples in H0-H48 group, n = 37 in D3 group, n = 10 in M1 group, n = 95 in M6-M12 group; flow cytometry: n = 4 samples in healthy donors group, n = 34 to 40 samples in H0-H48 group, n = 5 to 8 samples in M1 group. *p < 0.05, ****p < 0.001.

Since immunomodulatory effects of P2Y11 were previously described^21^, we evaluated the expression of its gene (*P2RY11*) and its potential variability in PBMC from patients after AMI. *P2RY11*gene expression increased in the first 48 h and then returned to baseline (fold-change = 1.72 for H0–H48 group vs. 1 for healthy donors, *p* = 0.0006) (Fig. 5).

Analyzing protein expression by flow cytometry, we observed a significant increase in CD3 + cells (median MFI = 751.8 for H0–H48 group vs. 180.8 for M1 group, *p* = 0.0143) and CD19 + cells (median MFI = 193.0 for H0–H48 group vs. 85.9 for healthy donors, *p* = 0.0206). We did not observe any significant variation of P2Y11 protein expression in CD45 + CD14 + CD11c + monocytes (Fig. 5).

### PBMC collected within the first two days after AMI induce a phenotype modification in human cardiac fibroblasts (HCF)

**Figure 6:**
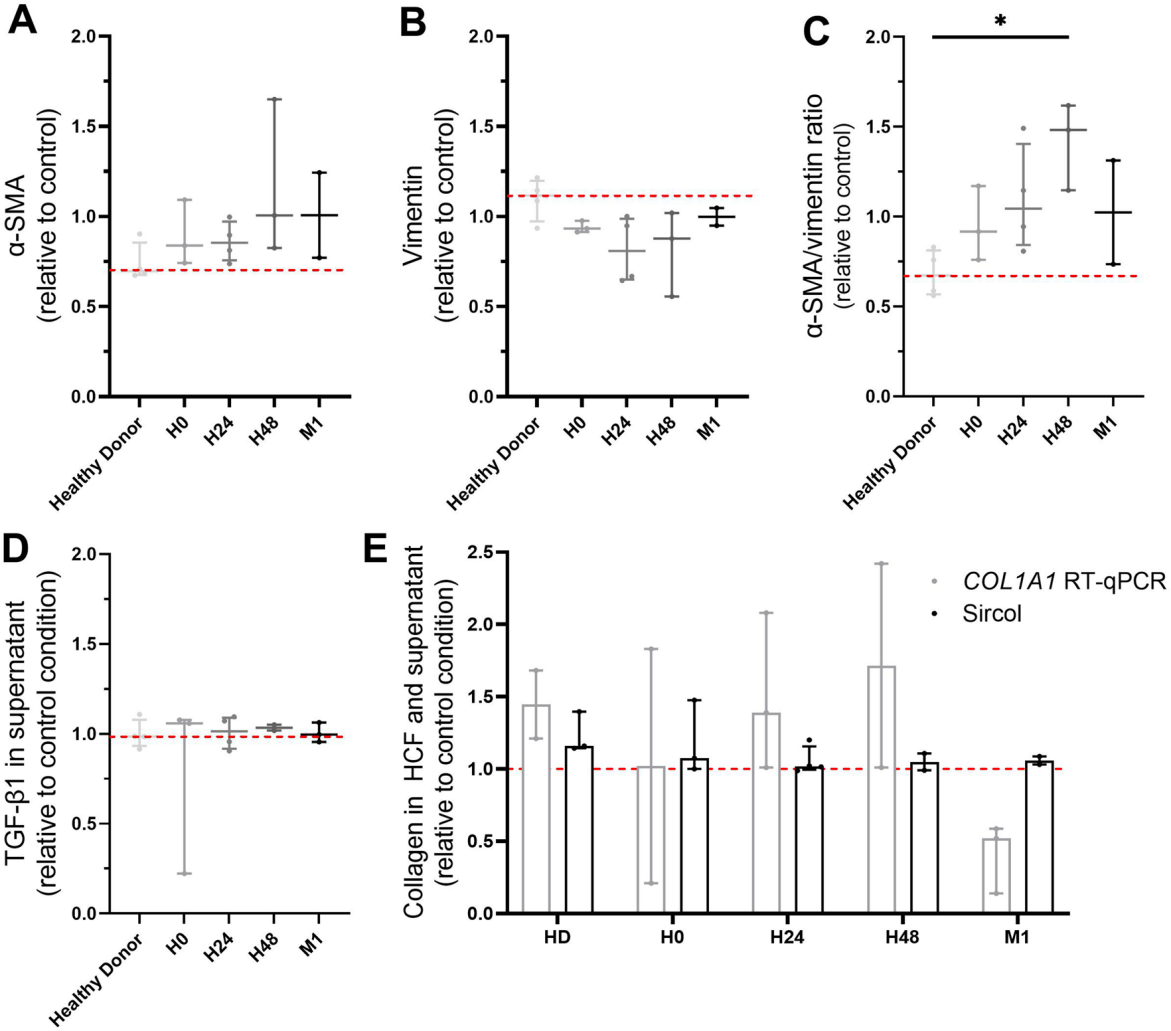
Characterization of cardiac fibroblasts after coculture with PBMC from collected at H0, H24, H48, M1 after myocardial infarction. (**A**) α-SMA, (**B**) vimentin, and (**C**) α-SMA/vimentin ratio by flow cytometry; (**D**) TGF-β1 secretion in supernatant by ELISA; (**e**) Collagen secretion in supernatant by Sircol Assay. n = 4 samples in healthy donors, n = 3 samples in H0 group, n = 4 samples in H24 group, n = 3 samples in H48 group, n = 2 samples in M1 group. *p < 0.05.

To study the phenotypic of cardiac fibroblasts and their acquisition of contractile properties, α-SMA and vimentin expression were analyzed in HCF after 24 h of coculture with PBMC coming from AMI patients of healthy donors. Since TGF-β1 is a cytokine known to be involved in the myofibroblastic phenotypic switch, and collagen is secreted by myofibroblasts, their concentrations in coculture supernatant were analyzed (Fig. 6). Although α-SMA and vimentin analyzed independently showed modest changes (α-SMA “condition”/ “control” ratio = 0.7 with PBMC from healthy donors vs. 1 with H48 PBMC from patients, *p* = 0.2402 ; vimentin “condition”/ “control” ratio = 1.11 with PBMC from healthy donors vs. 0.88 with H48 PBMC from patients, *p* = 0.1473), the α-SMA/vimentin ratio was decreased in HCF cultured with PBMC from healthy donors whereas it was increased with PBMC from patients collected at H48 after AMI compared to control condition (0.67 for PBMC from healthy donors condition vs. 1.48 for H48 PBMC, *p* = 0.0496).

No significant variation of TGF-β1 secretion was observed in supernatant of HCF cultured with both PBMC from healthy donors and STEMI patients (“condition”/ “control” ratio = 0.99 with healthy donors vs. 1.03 with H48 PBMC, *p* = 0.9971). IL-4, IL-10, IL-12, TNF-α were not detected in supernatants.

We did not observe any significant variation of collagen secretion even at RNA transcript level in HCF (“condition”/“control” ratio = 1.44 with PBMC from healthy donors vs. 1.72 with STEMI H48 PBMC, *p* = 0.2902) and at protein level in the supernatants (“condition”/ “control” ratio = 1.16 with PBMC from healthy donors vs. 1.05 with STEMI H48 PBMC, *p* = 0.4659).

Finally, P2Y11 expression did not significantly vary at both transcriptional level (“condition”/ “control” ratio = 1.03 with PBMC from healthy donors vs. 1.42 with STEMI H48 PBMC, *p* = 0.1416) and protein level in HCF (“condition”/“control” ratio = 0.96 with PBMC from healthy donors vs. 0.94 with STEMI H48 PBMC, *p* = 0.3097) after 24 h of coculture with PBMC (see Supplementary Fig. S4 online).

These results, performed in a small number of coculture experiments, suggested that PBMC from STEMI patients collected within the first 48 h could have an effect on human cardiac fibroblasts impacting their cytoskeleton structure.

### P2Y11 expression does not significantly vary in PBMC after in vitro hypoxia

**Figure 7:**
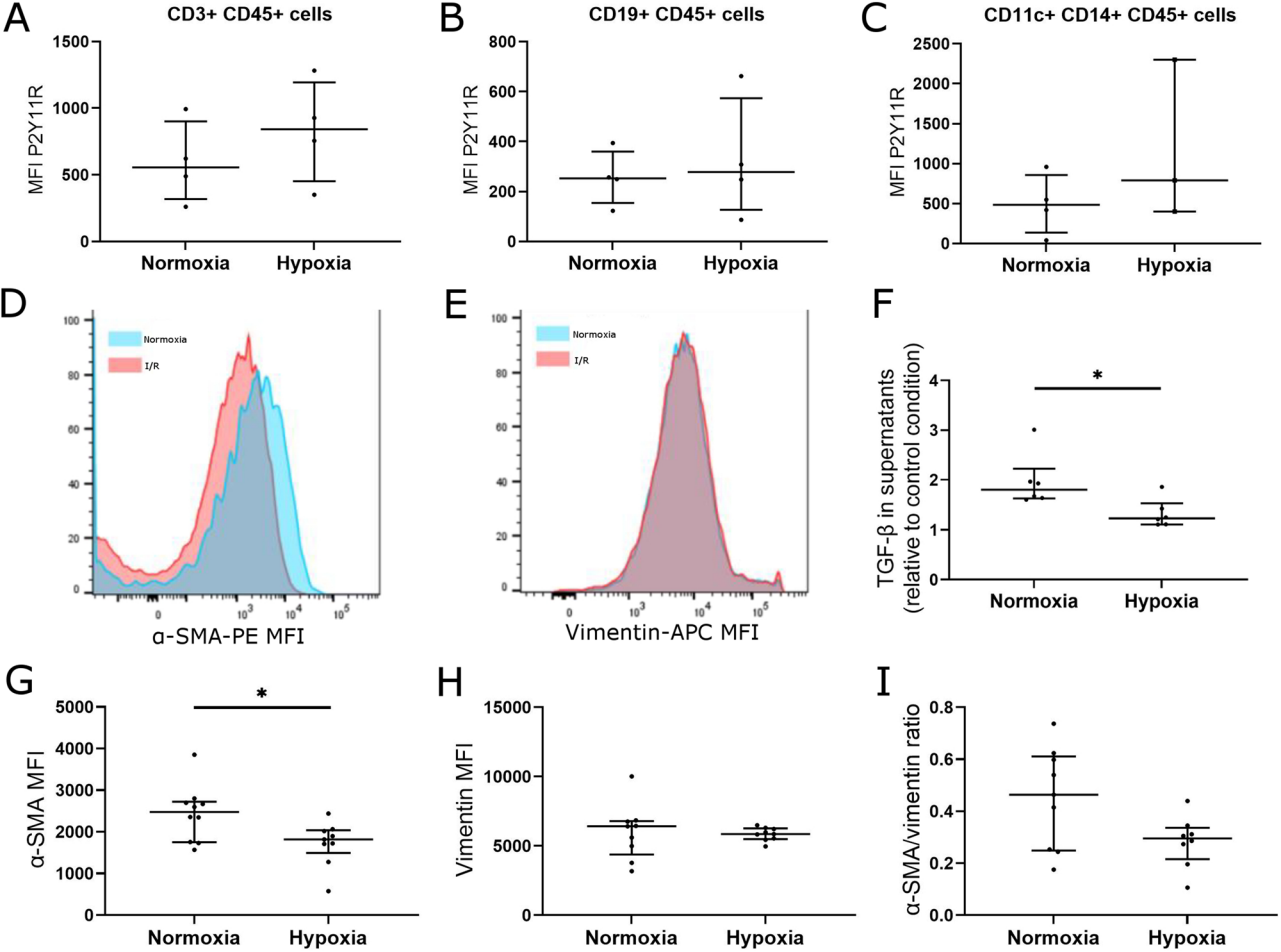
Effect of ischemia/reperfusion on P2Y11 expression in PBMC from healthy donors (**A**–**C**) and effect of conditioned supernatants on α-SMA, vimentin and TGF-β1 protein expression in cardiac fibroblasts by flow cytometry (d and e) and ELISA (f to i). P2Y11 MFI on (**A**) CD3 + cells, (**B**) CD19 + cells, and (**C**) CD14 + CD11c + cells. (**D**,**G**) α-SMA, (**E**,**H**) vimentin, (**I**) α-SMA/vimentin ratio, **F**) TGF-β1. P2Y11 expression: n = 4 samples in both groups, TGF-β concentration: n = 6 samples in both groups, α-SMA and vimentin expression: n = 8 samples in both groups. *p < 0.05.

We then assessed the effects of hypoxia (1% O_2_, 5 h) on the different populations of PBMC from healthy donors (CD3 + T cells, CD19 + B cells, CD11c + CD14 + monocytes) and did not observe any significant change in P2Y11 expression (P2Y11 MFI in CD3 +  = 555.5 vs. 841.5, *p* = 0.625; CD19 +  = 253.2 vs. 278.4, *p* = 0.875; CD14 + CD11c +  = 484.0 vs. 790.0, *p* = 0.25, for normoxia vs. hypoxia respectively) (Fig. 7A to E).

### Supernatants of PBMC subjected to hypoxia induce a decrease of α-SMA expression in human cardiac fibroblasts

We first studied the effect of hypoxia on the ability of PBMC to secrete TGF-β1 (Fig. 7F). As described, we studied TGF-β1 concentration in supernatants of PBMC subjected to either hypoxia or normoxia and compared it to TGF-β1 concentration in fresh medium (control condition). We observed that TGF-β1 concentration was increased in supernatant of PBMC cultured in normoxia as compared to control condition (ratio = 1.83, *p* = 0.0152) whereas it did not significantly vary in supernatant of PBMC subjected to hypoxia (ratio = 1.23).

This result suggested that PBMC lost their ability to secrete TGF-β1 under hypoxia condition.

We then studied the effect of mediators secreted by PBMC subjected to hypoxia on fibroblast activation. Therefore, α-SMA and vimentin expression were analyzed in HCF cultured during 24 h with conditioned supernatants obtained from PBMC subjected to either normoxia or hypoxia (Fig. 7). We observed a significant decrease in α-SMA expression in HCF cultured with supernatant of PBMC subjected to hypoxia (MFI = 2478 for normoxia condition vs. 1818 for hypoxia condition, *p* = 0.0278). Vimentin did not vary (6,421 for normoxia condition vs. 5,858 for hypoxia condition, *p* > 0.9999), and α-SMA/vimentin ratio tended to decrease in HCF cultured with conditioned supernatant of PBMC subjected to hypoxia (ratio = 0.46 for normoxia condition vs. 0.3 for hypoxia condition, *p* = 0.1094).

We did not observe significant differences in α-SMA or vimentin expression after fluorescence staining of HCF (see Supplementary Fig. S5 online).

## Discussion

The present study reports, for the first time, the temporal dynamic profile of inflammatory markers after AMI in two cohorts of STEMI patients with a good prognosis as defined by the preserved median left ventricular ejection fraction (55% at one year post-AMI). In this pilot study, peripheral mononuclear cells were shown to display an evolving profile with reparative characteristics and a low ability to secrete cytokines within the first two days after AMI. Moreover, these cells demonstrated the capacity to impact, in vitro, the expression of proteins involved in cardiac fibroblasts cytoskeleton structure, through the release of soluble mediators.

The immune response after AMI has largely been studied in rodents, leading to a comprehensive three-stage model. During the inflammatory phase, neutrophils and monocytes are recruited to the infarcted site, leading to secretion of pro-inflammatory cytokines and allowing the clearance of cellular debris^27^. Main CD4 + T cells found in the myocardium are Th1 cells, and Th2 are almost absent in the injured tissue^28,29^. The amount of Treg then increases and reaches a peak over the course of the reparative phase, while monocytes/macrophages switch towards an anti-inflammatory profile^3,4,6,9,16,30^. In human, some studies reported the association between peripheral Th1/Th2 ratio or plasma IL-6 levels and the severity of coronary artery disease but the inflammatory response has been less characterized^15–31^. Our study, performed in a cohort of patients with a favorable cardiac outcome, shows that the main variations were observed the first two days after AMI with a decrease of Th1/Th2 ratio and an upregulation of P2Y11R and of expression levels of genes involved in the regulation of dendritic cells tolerogenicity *STAT3* and *HMOX1* levels. From the third day, most of these parameters returned to baseline, suggesting that the activation of immune cells is transient, and that regulation of inflammation occurs early after the reperfusion in human.

STAT3, constitutively expressed in most cells, is induced by several factors, including IL-6 and IL-10 cytokines or ischemia^25,32^. As a transcription factor, it regulates various genes, notably anti-apoptotic pathway genes, and its role in inflammatory processes as observed after AMI, may be either pro- or anti-inflammatory depending on the environment and pathological context^32^. In myocardial infarction condition, its constitutive overexpression in transgenic mice is associated with a smaller infarct size in favor of a cardioprotective effect of STAT3^33^. HO1 is an intracellular enzyme which role is to catalyze heme oxidation, leading to generation of anti-inflammatory, anti-thrombotic and anti-apoptotic products. Various studies previously reported a protective effect of HO-1 induction against atherosclerosis progression, as well as its regulating effect on Th1/Th2 and Th17/Treg imbalances^34,35^. Interestingly, in our study which included patients with good outcome, both *HXMO1* and *STAT3* were overexpressed in PBMC in the early hours after AMI, in accordance with a protective effect of these proteins after AMI.

Contrarily to *STAT3* and *HMOX1*, we observed a decrease of *IDO1*, encoding for an intracellular enzyme responsible for catabolism of tryptophan and leading to production of kynurenine. Zara et al*.* previously reported a reduction of *IDO* mRNA in monocytes-derived dendritic cells of non-STEMI patients, and an increased activity of this pathway has been reported to be associated with a poorer prognosis in various cardiovascular diseases^36,37^. The present study provides additional arguments in favor of the downregulation of this enzyme in patients with a good outcome.

P2Y11 is a purinergic receptor ubiquitously expressed, activated by extracellular ATP, a danger-associated molecular pattern (DAMP) known to be massively released during myocardial infarction. The functions of this receptor are not yet fully understood however, several studies converge towards its anti-inflammatory effects. Chadet et al*.* showed, that under LPS stimulation, the activation of P2Y11 in dendritic cells inhibited IL-12 production, a cytokine involved in Th1 differentiation of T naïve cells, but preserved IL-10 secretion, a cytokine responsible for Treg differentiation. After a 5 h prolonged hypoxia, they observed a down-regulation of P2Y11 associated with a loss of its immunomodulatory effect^21^. Amisten et al*.* reported that the Thr-87 variant P2Y11 polymorphism was more frequent in acute myocardial infarction patients compared to control and associated with a higher CRP level^38^. In a murine model of cardiac allograft, Bourguignon et al*.* also observed that stimulation of a P2Y11-like receptor decreased allograft rejection and increased short-term graft survival^20^. Finally, Gruenbacher et al*.* reported a crosstalk between P2Y11 and TLR4 pathways and suggested that this receptor could be involved in the hyporesponsiveness of immune cells, after strong activation of TLR4 by LPS, leading to endotoxin tolerance phenomenon^39^. We can hypothesize that ischemia and the intense inflammatory response observed within the first hours after AMI may lead to an upregulation of P2Y11 in circulating immune cells thus modulating the systemic and local immune response and preventing the deleterious effects of an excessive inflammation.

PBMC collected during the first 24 h also displayed a decreased ability to secrete both pro- and anti-inflammatory cytokines (IL-6, IL-10, IFN-γ, TNF-α, IL-1β) after PMA/ionomycin stimulation. This represents a supplemental argument supporting the hypothesis that circulating T cells could develop an early hyporesponsiveness to additional stimuli after AMI leading to the regulation of pro-inflammatory cytokines secretion. In the context of an AMI, this phenomenon could prevent an extended inflammation and allow a long-term adapted ventricular remodeling, as suggested by the good outcome of our cohort. Moreover, this phenomenon may contribute to peripheral tolerance and prevent development of auto-immunity, particularly due to the release of cardiac-specific proteins like myosin^40–44^.

We also observed a CCL-2 preserved secretion after PMA/ionomycin stimulation in both early and lately collected PBMC. Even if the circulating level of CCL-2 at baseline has been previously reported to be associated with a higher cardiovascular risk, it also has an important role in wound healing^45^. In mouse, Dewald et al*.* demonstrated that CCL-2 is markedly secreted at the myocardial infarct site and associated with early recruitment of macrophages and accumulation of myofibroblasts^46^. Taken together, our results indicate that the preservation of CCL-2 secretion, in spite of loss of cytokine secretion ability during the first days, must be important for the reparative mechanisms.

It is speculated that an abnormally intense or prolonged inflammation after AMI may lead to a pathological healing and an extended fibrosis responsible for ischemic heart failure and its adverse clinical consequences. We therefore hypothesized that immune cells interact with cardiac fibroblasts and may modify their phenotype. We developed a contactless coculture model between PBMC from patients and human cardiac fibroblasts and observed that PBMC collected in the first two days that had a reparative profile seem to be able to modify some cytoskeleton proteins of cardiac fibroblasts represented by an increase of α-SMA/vimentin ratio. Interestingly, the use of PBMC from healthy controls had opposite effects, displaying a decrease of this ratio. Alpha-SMA is a thin filament found in the cytoplasm of smooth muscle cells and myofibroblasts, vimentin is an intermediate filament protein that is expressed in mesenchymal cells, including fibroblasts and myofibroblasts. Therefore, the modification of the α-SMA/vimentin ratio could indicate a higher degree of myofibroblast differentiation, which is associated with increased tissue stiffness, fibrosis, and reduced tissue elasticity. This modification in fibroblasts cytoskeleton could be due to secretion of soluble factors by PBMC. Among mediators known to activate the myofibroblastic transforming, TGF-β1 represents the major one, but other factors have been previously reported, such as CCN2 (Connective Tissue Growth Factor: CTGF) or cytokines such as IL-1, IFN-γ or TGF-β3^47^. In our model, TGF-β1 was not increased in the supernatant collected from coculture with PBMC from patients compared with that of healthy donors. Other cytokines such as IL-4, IL-12, IL-10, and TNF-α were not detected in the supernatant, suggesting that the observed proteins variations in fibroblasts may be due to other cytokines, and/or other soluble mediators, like non coding RNA for example^48^.

Finally, we hypothesized that, after a stress induced by in vitro hypoxia, immune cells could secrete mediators comparable to those released after AMI. Results obtained with conditioned supernatants showed an increase of TGF-β1 in the supernatants of PBMC in normoxia condition, whereas this concentration was similar in hypoxia conditioned supernatants compared to control, supporting that that hypoxia prevents PBMC from secreting TGF-β1. Moreover, contrary to coculture experiments, hypoxia conditioned supernatants of PBMC induced a decrease in α-SMA and, to a lesser degree, of α-SMA/vimentin ratio in cardiac fibroblasts. All these results suggest that AMI healing involves interactions between cardiac fibroblasts and PBMC that did not directly suffer from hypoxia in the myocardium but that are recruited from lymph nodes after their activation. These PBMC could secondarily circulate in the blood and participate to the modulation of both systemic and local inflammatory response and in the differentiation of fibroblasts at the site of infarction. This hypothesis is supported by the results of Hofmann et al*.* who observed that collagen deposition in mice heart after AMI requires the activation of CD4 + T cells in mediastinal lymph node^49^.

Most data dealing with inflammation post-AMI were collected from in vitro or murine models and their applicability to human remains questionable. If immunology and inflammation responses in human cardiac homeostasis and heart diseases has been a field of interest for two decades, it has also been limited due to their complexity and their variety as well as the restrained access to myocardium. In this context, a major limitation of our study stems from the analysis of peripheral inflammatory response only. However, only a very few studies investigated the transferability of peripheral data to cardiac tissue. For this purpose, Lluberas et al*.*^50^ compared distinct immune cells subsets and cytokines in the peripheral blood and at the site of the coronary occlusion. They reported that the amount of total T cells, CD4 + T, CD8 + and Treg cells was comparable in both samples. However, CD28^null^ CD4 + T cells, a subset of T cells with pro-inflammatory properties, as well as the number of IFN-γ and IL-10 producing CD4 + T cells were increased in intracoronary samples. This suggest that some pro-inflammatory subsets of PBMC are more likely to be found at the site of occlusion than in the peripheral blood, and that the peripheral may not be the exact reflect of the myocardial immune activity. Bönner et al*.* recently reported, using fluorine ^19^F cardiovascular magnetic resonance in pig model, that 6 days after a reperfused AMI, monocytes/macrophages ^19^F signal in myocardium was correlated to increase of circulating leukocytes^13^. Consequently, gathering these results with ours, we can suppose that these circulating immune cells represent an important subset of activated cells involved in communication with cardiac cells, particularly fibroblasts, and cardiac wound healing.

Other limitations of our work need to be acknowledged. First, our population of AMI patients is a rather low risk population with limited atherosclerotic risk factors (5% of diabetes, 38% of hypertension) and preserved left ventricular ejection fraction. One may hypothesize that the inflammatory response may be different in elderly patients and/or patients with severe comorbidities such as diabetes. Second, we have no clinical data regarding healthy donors whom blood samples were used in our experiment. One may argue that some may have unknown risk factors such as diabetes or low-grade chronic inflammation that may impact our results. Third, the variation of the number of samples in the different groups, particularly in the blood samples analyzes, is an issue that we had to manage and that may represent a limitation in the interpretation of the results. This variation is explained not only by the inclusion of patients that was inconstant in time throughout the study, but also by the limited amount of available PBMC per patient that did not allow us to perform all the experiments for all patients. Finally, a large number of PBMC is required for the coculture experiment to have the opportunity to reveal a potential temporal effect. This would require a large number of PBMC, that could be obtain pooling PBMC subtypes from several patient samples. However, we provided a pilot study with a human cohort of good prognosis, laying the foundations for the comprehension of reparative mechanisms in optimal conditions and opening the door to broader perspectives.

We conclude that inflammation and reparative processes after AMI are complex and require various factors including cytokine secretion, immune cell-polarization and cell-interactions. Immune response is a dynamic process that vary according to sites and time after AMI. Our study provides a new insight into the human pathophysiology of inflammatory response after myocardial infarction and its potential impact on fibroblasts mediated fibrosis and remodeling.

**Figure 8:**
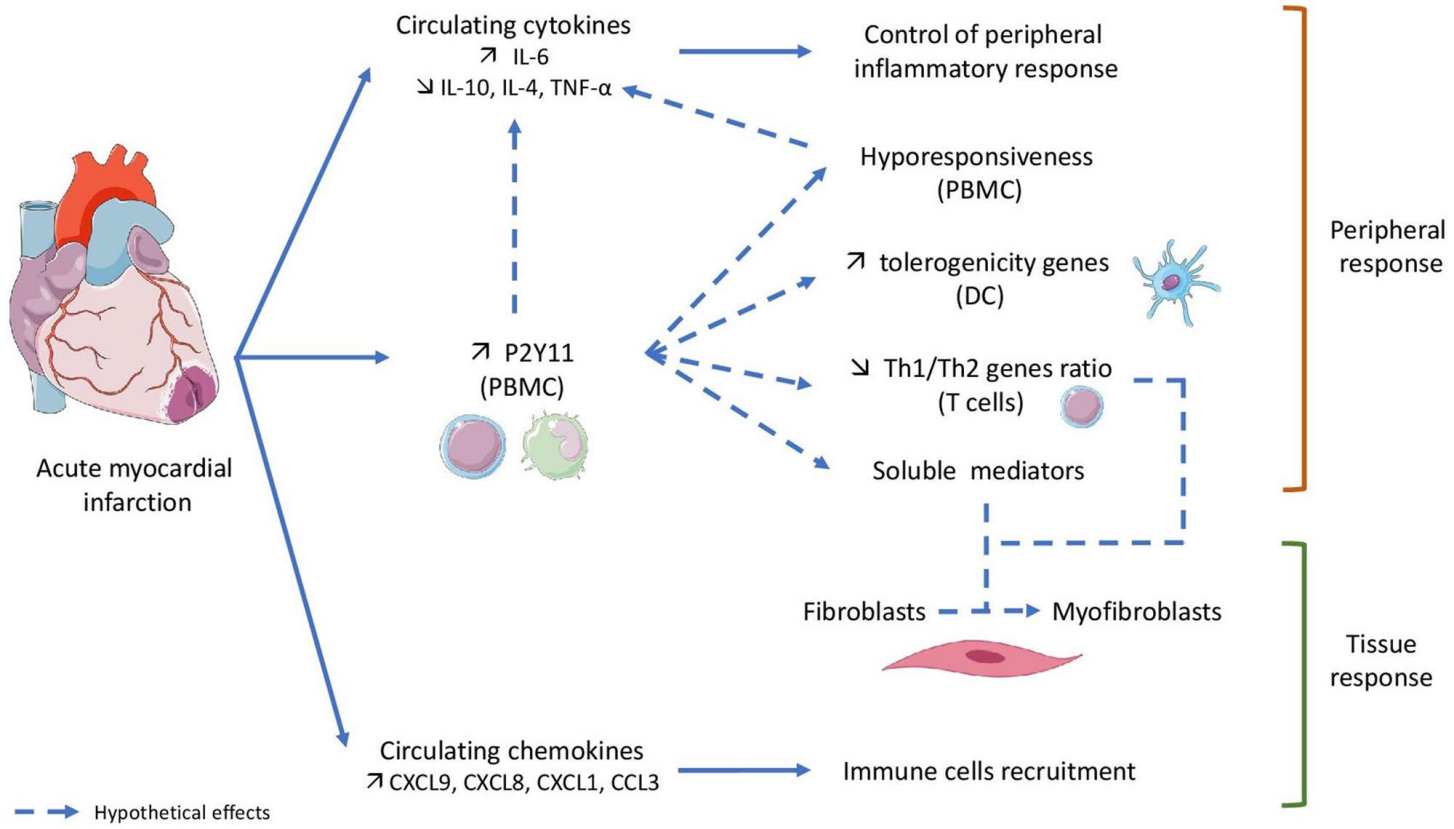
Acute myocardial infarction (AMI) is responsible for an increase of P2Y11 expression in PBMC that could lead to a modification of immune cells transcriptional profile resulting in a tolerogenic polarization of dendritic cells and a decrease of Th1/Th2 ratio. P2Y11 receptor could also be involved in PBMC hyporesponsiveness early after AMI and, as a consequence in cytokines and chemokines secretion changes. This immune peripheral response may partly explain the tissue response including differenciation of fibroblasts in myofibroblasts. P2Y11 represents a cornerstone of the control of inflammatory response after myocardial infarction.

In our point of view, P2Y11 may represent a cornerstone of the control of inflammatory response after myocardial infarction and our study allowed us to observe a temporal variation of this receptor in peripheral immune cells. The present study represents a preliminary work which aim was to describe the presence or not of a variation of this immune-inflammatory response and of this receptor in human immune cells after MI, but it also raises several questions regarding its active role in immune cells polarization and in interactions with cardiac fibroblasts (Fig. 8).

## Materials and methods

### Cohorts inclusion criteria

Blood samples of patients admitted at the acute phase of ST-segment elevation myocardial infarction (STEMI) were collected at the Cardiology department of University Hospital of Tours, between January 2019 and September 2021. These patients were enrolled in two prospective cohorts: Hibiscus-STEMI (coHort of patients to Identify Biological and Imaging markerS of CardiovascUlar outcomes in ST elevation myocardial infarction, NCT03070496) and CARIM (CARdioprotection in Myocardial Infarction, NCT02967965). Inclusion criteria were: first STEMI requiring a primary percutaneous coronary intervention, age 18–90 years, onset of chest pain of less than 12 h, informed consent. Exclusion criteria were: diagnosis of STEMI not confirmed by angiography, refusal to participate in the study or to sign the consent, impossibility to give information to the subject about the study, lack of medical social coverage, obvious contraindication to magnetic resonance imaging (claustrophobia, pacemaker, defibrillator, renal insufficiency, known allergy to a contrast agent), deprivation of civil rights, participation to another interventional study. Informed consent was obtained from all subjects. Experimental protocols were approved by the Comité de Protection des Personnes Sud-Est IV (ref 2012-A00313-40) and Comité de Protection des Personnes Sud-Est III (ref 2016-A01645-46) for CARIM and Hibiscus-STEMI cohorts respectively, and were performed in line with the principles of the Declaration of Helsinki.

### Peripheral blood mononuclear cells (PBMC) and plasma collection

Blood samples (2 tubes of 10 mL) from patients were collected at different times after myocardial infarction: at the time of angiography (H0), and 4 h (H4), 24 h (H24), 48 h (H48), 1 month (M1) after reperfusion for the Hibiscus-STEMI cohort, and 3 days (D3), 6 months (M6), 12 months (M12) after reperfusion for the CARIM cohort. Blood samples from healthy donors were obtained by the Etablissement Français du Sang (French blood transfusion agency).

Even if collected after AMI, PBMC cultured without stimulation did not secrete the cytokines of interest (IL-1β, IL-6, IL10, IFN-γ, TNF-α). Cells were then stimulated using phorbol myristate acetate (PMA) and ionomycin. Fresh PBMC were deposited in 2 wells of a 96-well plate (2 × 10^5^ PBMC/well) with 200µL of Gibco™ RPMI (Roswell Park Memorial Institute medium, ThermoFisher Scientific, Waltham, MA, USA) complete medium (RPMI with 10% FBS, and 1% penicillin–streptomycin solution), and incubated with (condition well) or without (control well) PMA and ionomycin at a final concentration of 50 ng/mL and 1 µg/mL respectively. Supernatants were collected after overnight incubation and stored at − 20 °C until use.

Plasma was collected after tubes centrifugation (800 g for 12 min) and PBMC using Lymphosep™ (Lymphocyte separation medium, Dutscher, Belgium), and frozen in 90% fetal bovine serum (FBS) and 10% DMSO at − 80 °C after isolation.

### Analyses of cytokines and chemokines from plasma by multiplex flow cytometry

Plasma chemokines and cytokines were analyzed in a small subset of healthy donors and patients using respectively LEGENDplex™ Human Proinflammatory Chemokine panel and LEGENDplex™ HU Essential Immune Response (Biolegend, San Diego, CA, USA), and following manufacturer’s instructions. Samples were analyzed using MACSQuant Analyzer (Miltenyi Biotec, Bergisch Gladbach, Germany). The results are presented as median of concentration in pg/mL for each group.

### Flow cytometry

To evaluate immune and P2Y11 profile of PBMC from patients and healthy donors, fresh PBMC were incubated in a 96-well plate (5 × 10^5^ PBMC/well) 1µL of CD45-APC-H7, CD3-BV786, CD19-BB700, CD11c-VB et CD14-PE (respective clones: 2D1, SK7, SJ25C1, REA618, REA599, BD Biosciences, San Jose, CA) antibodies during 20 min at 4 °C (see Table 2 for the complete list of antibodies and dies used for flow cytometry). After washing in PBS, fixation and permeabilization was performed during 20 min at 4 °C (BD Cytofix/Cytoperm™ Fixation/Permeabilization Solution Kit, 50µL, BD Biosciences, San Jose, CA, USA), and cells of one well were incubated with 25 µL of anti-human P2Y11 receptor primary antibody (APR015, rabbit anti-human, 1:500, Alomone Labs, Jerusalem, Israel) during 30 min at 4 °C. Cells of another well were incubated with 25µL of both anti-P2Y11 antibody and P2Y11 peptide (1:500) during 30 min at 4 °C (negative control). After washing in Perm Buffer, cells from the two wells were incubated with 100 µL of AF647 secondary antibody (goat anti-rabbit, 1:1000, ThermoFisher Scientific, Waltham, MA, USA) during 45 min at 4 °C.

**Table 2:** Antibodies and dies for flow cytometry.

Antibodies	Fluorochrome	Clone	Concentration	Manufacturer
CD45	APC-H7	2D1	Undiluted	BD Biosciences
CD3	BV786	SK7	Undiluted	BD Biosciences
CD19	BB700	SJ25C1	Undiluted	BD Biosciences
CD11c	VioBlue	REA618	Undiluted	BD Biosciences
CD14	PE	REA599	Undiluted	BS Biosciences
P2Y11 receptor primary antibody		APR015	1:500	Alomone Labs
Goat anti-rabbit secondary antibody	AF647		1:1000	ThermoFisher Scientific
Viobility™ 405/520 fixable dye			Undiluted	Miltenyi Biotec
Goat anti-rabbit secondary antibody	AF405		1:250	ThermoFisher Scientific
Alpha-smooth muscle actin	PE	1A4	Undiluted	Bio-techne
Vimentin	APC	REA409	Undiluted	Miltenyi Biotec

To assess the effects of supernatant from PBMC after hypoxia and of coculture with PBMC in differentiation of fibroblasts into myofibroblasts during hypoxia and coculture experiments, HCF were first incubated with 1 µL of Viobility™ 405/520 fixable dye (Miltenyi Biotec, Bergisch Gladbach, Germany) during 10 min at 4 °C, and then permeabilized (BD Cytofix/Cytoperm™ Fixation/Permeabilization Solution Kit, BD Biosciences) and incubated with 25 µL of primary anti-P2Y11 antibody and 1 µL of anti-vimentin-APC (clone REA409, Miltenyi Biotec, Bergisch Gladbach, Germany) during 30 min at 4 °C. Cells were then stained with 50 µL of anti-P2Y11 AF405 secondary antibody (goat anti-rabbit, 1:250, ThermoFisher Scientific, Waltham, MA, USA) and 2 µL of anti-alpha-smooth muscle actin-PE (clone 1A4, Bio-techne, Mineapolis, MI, USA) during 1 h at 4 °C.

Cell staining was analyzed using the FACSMelody™ cell sorter, and results were analyzed with FlowJo™ software. The median of fluorescence intensity (MFI) was obtained for each staining. Results are presented as the percentage of CD45 + cells for CD3, CD19, CD11c, CD14 staining, as Mean Fluorescence Intensity (MFI) for P2Y11 expression in PBMC, and for P2Y11, α-SMA and vimentin for hypoxia experiments. For coculture experiments, to study the effect of PBMC, results were expressed as the ratio of “PBMC well”/ “control well”. Median of these results was obtained for each group.

### RNA extraction, reverse transcription and real-time relative quantitative PCR

PBMC remaining after flow cytometry and stimulation experiments were centrifuged to obtain a pellet that was stored at − 20 °C until use. RNA extraction was performed with TRIzol™ reagent according the manufacturer’s instructions (ThermoFischer Scientific, Waltham, MA, USA). RNA was resuspended in 25 µL of RNase free water, and concentration and purity were determined using a NanoDrop™ spectrophotometer (ThermoFisher Scientific, Waltham, MA, USA). Reverse transcription was performed using PrimeScript® RT Reagent kit (Takara, Shiga, Japan) and Flexlid thermocycler (Eppendorf, Hamburg, Germany) following Takara’s instructions. cDNA obtained were stored at − 20 °C.

To study various genes in PBMC previously reported to be involved in tolerogenicity profile of dendritic cells^25^, as well as genes involved in T CD4 + cells polarization, and *P2RY11* gene we performed a qPCR using TB green® Premix ExTaq (Takara) in a CFX ConnectReal Time System (Bio-Rad) following Takara’s recommendation (see Table 3 for primers sequences). *PPIA* was used as reference gene after validation with NormFinder^26^. For each group, results were expressed as median of fold-change using 2^−ΔΔCt^ method where Ct represents the cycle threshold and ΔΔCt = [(Ct target gene − Ct reference gene) patient] − [median (Ct target gene − Ct reference gene) healthy donors].

**Table 3:** Primers sequences for qPCR, F for forward and R for reverse primers.

Gene	Primers sequences
*PPIA*	PPIA-F: 5′-ACCGCCGAGGAAAACCGTGTA-3′
	PPIA-R: 5′-TGCTGTCTTTGGGACCTTGTCTGC-3′
*STAT3*	STAT3-F: 5′-GGAACAAGCCCCAACCGGA-3′
	STAT3-R: 5′-CTAAAATCAGGGGTCCCAACTGT-3′
*HMOX1*	HMOX1-F: 5′-ACCCATGACACCAAGGACCAGA-3′
	HMOX1-R: 5′-GTGTAAGGACCCATCGGAGAAGC-3′
*IDO1*	IDO-F: 5′-TCATCTCACAGACCACAA-3′
	IDO-R: 5′-GCAGTAAGGAACAGCAATA-3′
*P2RY11*	P2RY11-F: 5′-TAGCAGACACAGGCTGAGGA-3′
	P2RY11-R: 5′-CACCAGGAACTCAACCACCA-3′
*TBX21*	TBX21-F: 5′-GCCTACAGAATGCCGAGATTACT-3′
	TBX21-R: 5′-GGATGCTGGTGTCAACAGATG-3′
*FOXP3*	FOXP3-F: 5′-GAACGCCATCCGCCACAACCTGA-3′
	FOXP3-R: 5′-CCCTGCCCCCACCACCTCTGC-3′
*GATA3*	GATA3-F: 5′-TCA TTA AGC CCA AGC GAA GG-3′
	GATA3-R: 5′-GTC CCC ATT GGC ATT CCT C-3
*CD8*	CD8-F: 5′-CAGGCCAGAGACCCAGAA-3′
	CD8-R: 5′-GAAACCAGCAGAACCAGGAC-3′
*CD4*	CD4-F: 5′-TGGCTCTGGAAACCTCACCCT-3′
	CD4-R: 5′-GGCACTGGCAGGTCTTCTTCT-3′
*RORC*	RORC-F: 5′-GCAGCGCTCCAACATCTTCT-3′
	RORC-R: 5′-ACGTACTGAATGGCCTCGGT-3′
*HPRT1*	HPRT1-F: 5′-TTGCTGACCTGCTGGATTAC-3′
	HPRT1-R: 5′-TATGTCCCCTGTTGACTGGT-3′
*ACTA2*	ACTA2-F: 5′-ACCCGATAGAACATGGCATC-3′
	ACTA2-R: 5′-CATACATGGCTGGGACATTG-3′
*VIM*	VIM-F: 5′-GTTTCCAAGCCTGACCTCAC-3′
	VIM-R: 5′-TTCCAGGGACTCATTGGTTC-3′
*COL1A1*	COL1A1-F: 5′-GATTCCCTGGACCTAAAGGTGC-3′
	COL1A1-R: 5′-AGCCTCTCCATCTTTGCCAGCA-3′

To study expression levels of *ACTA2*, *VIM*, *COL1A1* gene in cardiac fibroblasts after coculture with PBMC, we also performed a qPCR using TB green® Premix ExTaq (Takara). *HPRT1* was used as reference after validation with NormFinder (26). For each experiment, results were expressed as median of fold-change using 2^−ΔΔCt^ method where ΔΔCt = [(Ct target gene − Ct reference gene) condition with PBMC] − [median (Ct target gene − Ct reference gene) control without PBMC].

### ELISA assay

For PBMC stimulation experiments, IL-6, IL-10, IFN-γ, TNF-α, IL-1β cytokines and CCL-2 chemokine concentrations were measured in supernatants; for both hypoxia and coculture experiments, IL-4, IL-12, IL-10, TNF-α, TGF-β1 concentrations were measured in supernatants using ELISA kits (Human IL-1β, IL-4, IL-6, IL-10, IL-12, IFN-γ, TNF-α Uncoated ELISA and Human/Mouse TGF beta 1 Uncoated ELISA, ThermoFisher Scientific, Waltham, MA, USA) and following manufacturer instructions. An acidification of samples with hydrochloric acid (1N, 40µL) was performed for TGF-β1 concentration measurement.

For hypoxia experiments, results were expressed as ratios of the concentration of TGF-β1 in supernatant (after hypoxia or normoxia) to that of the control condition corresponding to the concentration of TGF-β1 in fresh medium, not used to culture PBMC in normoxia/hypoxia experiments. For coculture experiments, results were expressed as ratios of “PBMC wells” on that of “control wells” containing no PBMC. Medians were obtained for each group.

### Sircol assay

Soluble collagen concentration was analyzed in supernatant of human cardiac fibroblasts cocultured with PBMC, following manufacturer instructions. Briefly, 100µL of supernatant was incubated with 500 µL of Sircol Dye reagent during 30 min under slow agitation. After centrifugation (12,000 rpm, 10 min), supernatant was removed and 750 µL of Acid Salt Wash reagent was put on the pellet before centrifugation (12,000 rpm, 10 min). Supernatant was removed and 250 µL of Alkali reagent was added to dissolve the pellet. Experiment was performed in duplicate for each sample. Finally, 200µL of each tube was transferred in a 96-well plate and the microplate reader was set to 540 nm to measure absorbance. Results were expressed as ratios of “PBMC wells” on that of “control wells” containing no PBMC. Medians were obtained for each group.

### Cocultures of human cardiac fibroblasts with PBMC

Cell cultures were performed using primary human cardiac fibroblasts (HCF) (HCF-av, ScienCell Research Laboratories, San Diego, CA, USA) (3 to 5 passages) and PBMC collected from healthy donors and from patients at different times after myocardial infarction (H0, H24, H48, M1). According to the manufacturer’s instructions, HCF were cultured with complete medium (Fibroblast Medium 2 (FM-2) with 5% FBS, 1% Fibroblast Growth Supplement-2 (FGS-2), and 1% penicillin–streptomycin solution, ScienCell Research Laboratories, San Diego, CA, USA) in a 37 °C, 5% CO_2_ atmosphere.

The first day, 3 × 10^5^ HCF were seeded in a 6-well plate and grown in FM-2 complete medium. PBMC were thawed and cultured with Gibco™ RPMI (Roswell Park Memorial Institute medium, ThermoFisher Scientific, Waltham, MA, USA) complete medium (RPMI with 10% FBS, and 1% penicillin–streptomycin solution) in a separate 6-well plate during 24 h in the incubator. The medium of HCF was then replaced by DMEM complete medium, and inserts with 0.4 µm pores were placed in each well. 1.5 × 10^6^ PBMC were distributed in the upper chamber of inserts. For each insert containing PBMC (“PBMC well”), a “control well” (insert without PBMC) was performed. After 24 h of coculture, supernatants were collected and stored at − 20 °C, and fresh HCF were stained and analyzed by flow cytometry.

### Experiments under hypoxic conditions

PBMC from healthy donors were distributed at the density of 1 × 10^6^ per well in 6-well plates. For the “normoxia” condition, PBMC were placed in the incubator (21% O_2_, 5% CO_2_, 37 °C) with DMEM (Dubelcco’s Modified Eagle Medium, ThermoFisher Scientific, Waltham, MA, USA) complete medium (5% FBS, and 1% penicillin–streptomycin solution) during 5 h, then medium was replaced by fresh complete medium and cells were cultured during 24 h in similar conditions (21% O_2_, 5% CO_2_, 37 °C). In the “hypoxia” condition, in order to simulate both acute oxygen and nutrient deprivation as observed in acute myocardial ischemia, PBMC were placed in the hypoxic workchamber in PBS containing Ca^2+^ and Mg^2+^ (1% O_2_, 5% CO_2_, 37 °C) during 5 h. For the reperfusion, in order to simulate acute myocardial reperfusion following successful coronary revascularization, PBS was replaced by DMEM complete medium and cells cultured in the incubator during 24 h (21% O_2_, 5% CO_2_, 37 °C). Supernatants from these two conditions were collected at the end of the experiments and HCF (3 × 10^5^ HCF in each well of a 6-well plate) were cultured with the conditioned medium in an incubator (21% O_2_, 5% CO_2_, 37 °C). After 24 h, HCF were collected and their expression of markers were analyzed by flow cytometry.

#### Statistical analyses

Data obtained from analyses performed from plasma and PBMC collected from H0 to H48 after AMI were pooled together (H0–H48), as well as samples collected from M6 to M12 (M6–M12) after AMI. The rank based nonparametric Kruskal–Wallis test was used to compare results from three or more groups of samples, followed by a Mann–Whitney test between groups when Kruskall–Wallis test showed statistically significant differences between two or more groups. Differences were considered significant at *p* < 0.05. Statistical analyses and figures were performed with GraphPad Prism software (GraphPad Software, San Diego, CA, USA).

### Supplementary Information

Supplementary Information.

## Data Availability

The datasets are available from the corresponding author on reasonable request.
