# Supplementary material for: Human peripheral blood mononuclear cells display a temporal evolving inflammatory profile after myocardial infarction and modify myocardial fibroblasts phenotype
Source: Sci Rep. 2023 Oct 5;13:16745. doi: 10.1038/s41598-023-44036-3 (PMC10556078; doi:10.1038/s41598-023-44036-3)
Supplement: Supplementary file 1 — Supplementary Information. [file 41598_2023_44036_MOESM1_ESM.docx]

**Supplementary data**

**Supplementary Figure 1**: Chemokines (a to d) and cytokines (e and h) concentration (pg/mL) in plasma of healthy donors and patients after myocardial infarction (non-pooled data). n=3 to 5 in healthy donors group, n=2 to 5 in each patients group. (a) CXCL9 (MIG), (b) CXCL8 (IL-8), (c) CXCL1 (GRO-α), (d) CCL3 (MIP-1α), (e) IL-4, (f) TNF-α, (g) IL-6, (h) IL-10. Error bars correspond to interquartile range. Kruskall-Wallis test was used to compare the 8 groups and the Mann-Whitney rank sum test to compare 2 groups. * p<0.05, ** p<0.01.


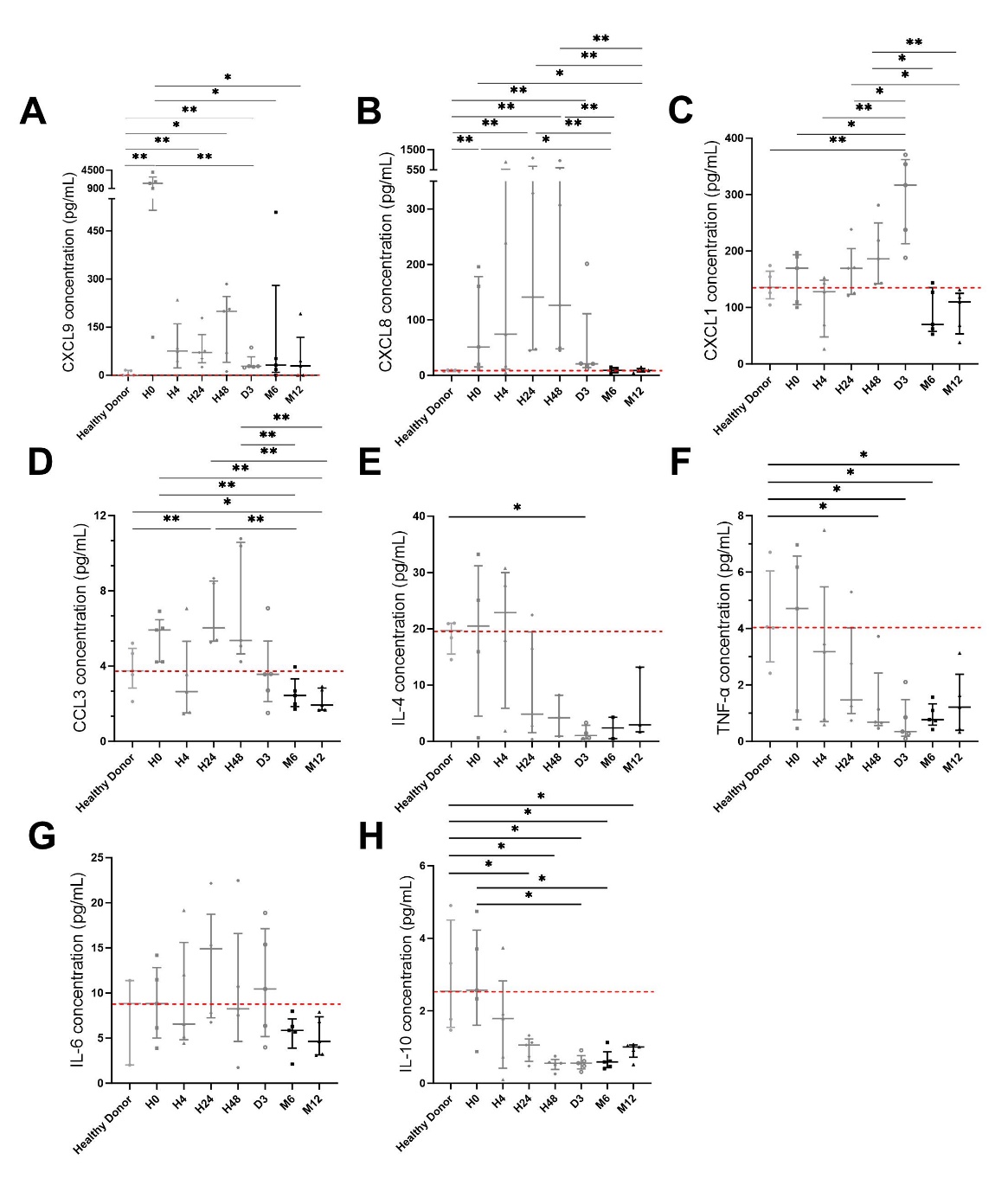


**Supplementary Figure 2**: Cytokines concentration (pg/mL) in plasma of healthy donors and patients after myocardial infarction. n=4 in healthy donors group, n= 16 to 20 in H0-H48 group, n=2 to 5 in D3 group; n=7 to 10 in M6-M12 group for IL-2, IL-1β, IFN-γ, IL-12 ; n=2 in healthy donors group, n=2 in H0-H48 and D3 groups, n=1 in M6-M12 group for TGF-β1. (a) IL-2, (b)IL-1β, (c) IL-17, (D) IFN-γ, (E) IL-12, (F) TGF-β1. Error bars correspond to interquartile range. Kruskall-Wallis test was used to compare the 4 groups.


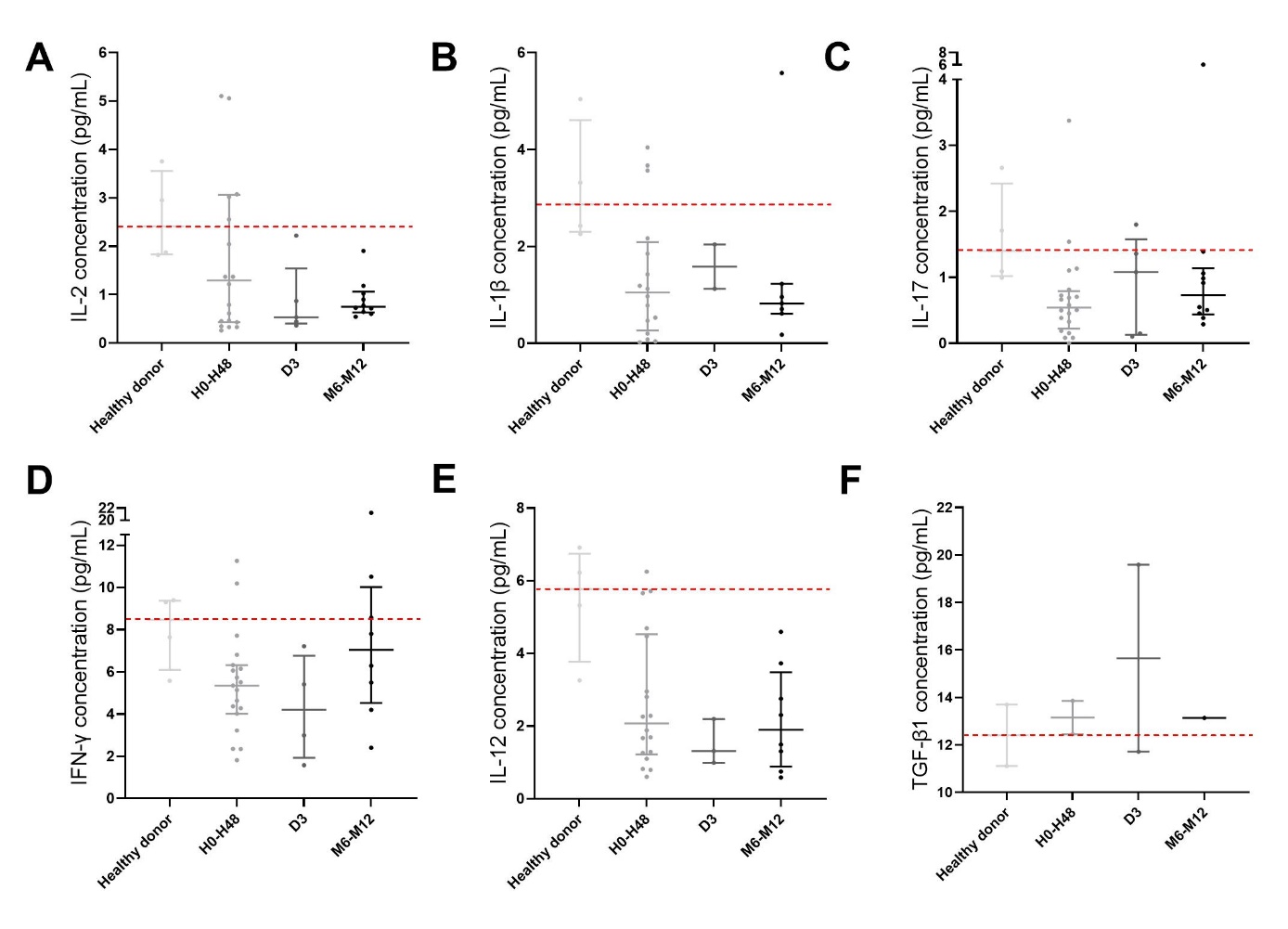


**Supplementary Figure 3**: Expression levels of genes involved in tolerogenicity profile of dendritic cells (a to c) and of T cells genes (d to i). (a) *HMOX1*, (b) *STAT3*, (c) *IDO1*, (d) *CD3*, (e) *CD4*, (f) *CD8*, (g) *TBX21*/*GATA3* ratio, (h) *FOXP3*, (i) *RORC*. n=14 to 17 in healthy donors group, n= 11 to 13 in H0 group, n=8 to 9 in H4 group, n=11 to 12 in H24 group, n=7 to 10 in H48 group, n=18 to 36 in D3 group, n=9 to 10 in M1 group, n=34 to 50 in M6 group, n=30 to 47 in M12 group. Error bars correspond to interquartile range. Kruskall-Wallis test was used to compare the 4 groups, Mann-Whitney rank sum test to compared 2 groups. * p<0.05, ** p<0.01, *** p<0.005, **** p<0.0001

**
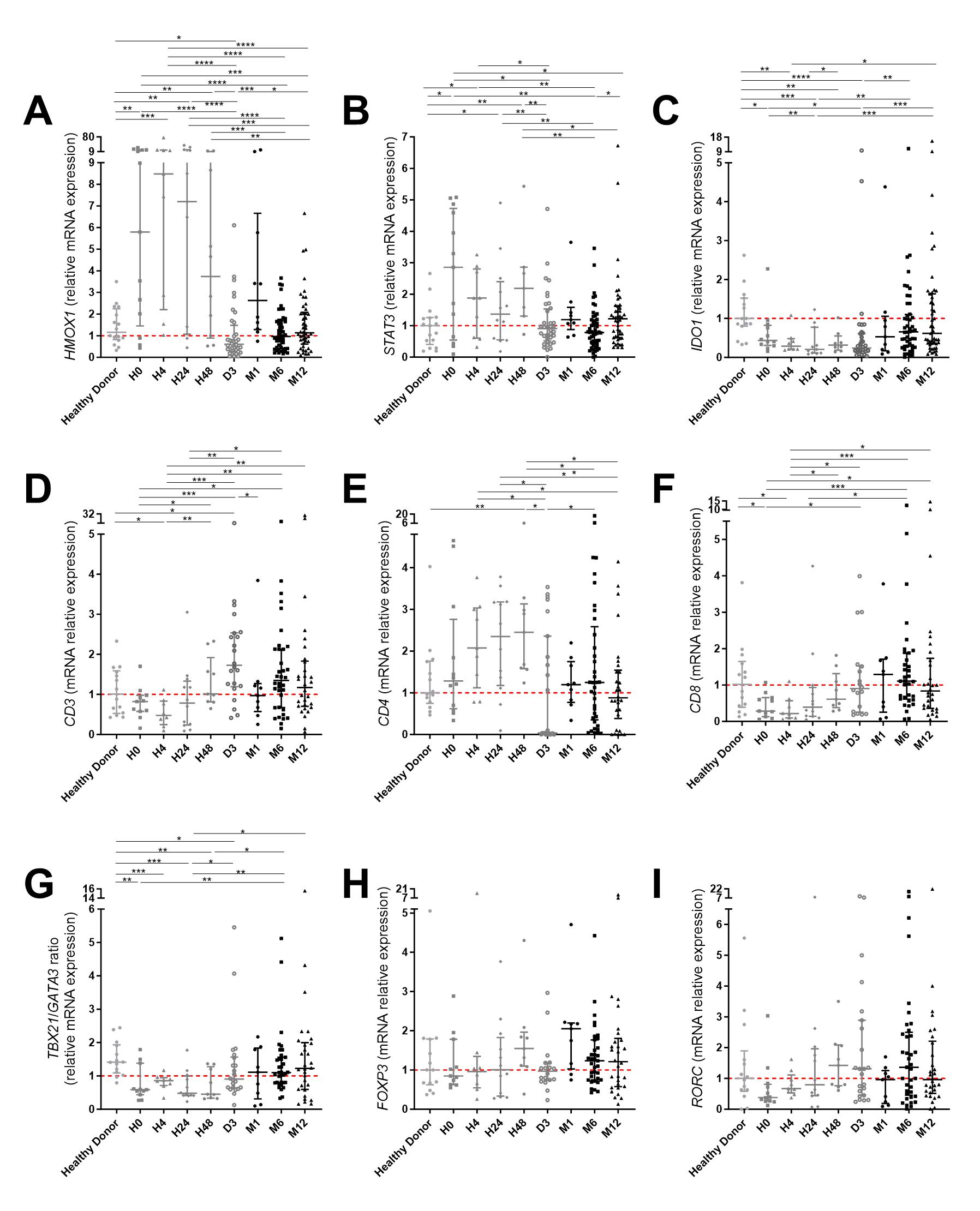
**

**Supplementary Figure 4:** Characterization of P2Y11 receptor expression in cardiac fibroblasts after coculture with PBMC from collected at H0, H24, H48, M1 after myocardial infarction by RT-qPCR and flow cytometry. n=2 to 4 in healthy donors group, n=2 to 3 in H0, H48 and M1 groups, n=3 to 4 in H24 group.


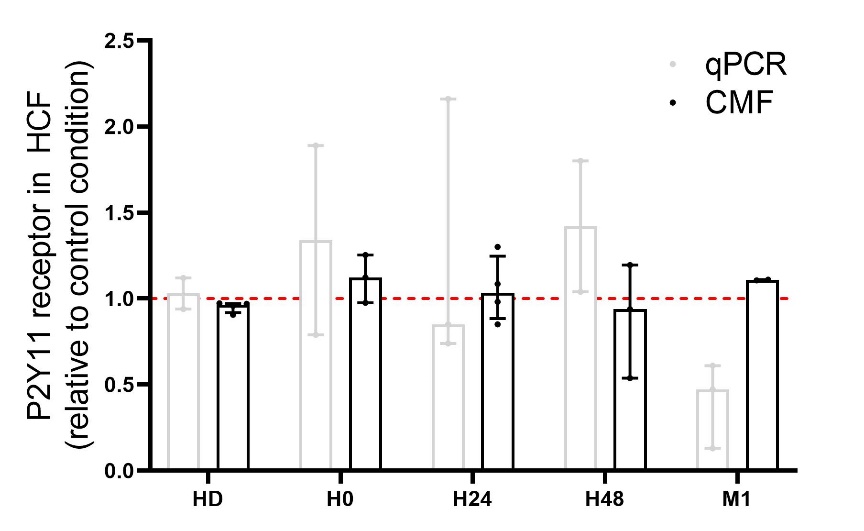


**Supplementary Figure 5:** F-actin (red, phalloidin staining) and α-SMA (green) staining of cardiac fibroblasts (DAPI stains the nuclei in blue) after 24hrs of culture with conditioned supernatants. Supernatants were obtained from PBMC after normoxia (a) or hypoxia/reoxygenation (b) condition. Scale: 50μM. X60.


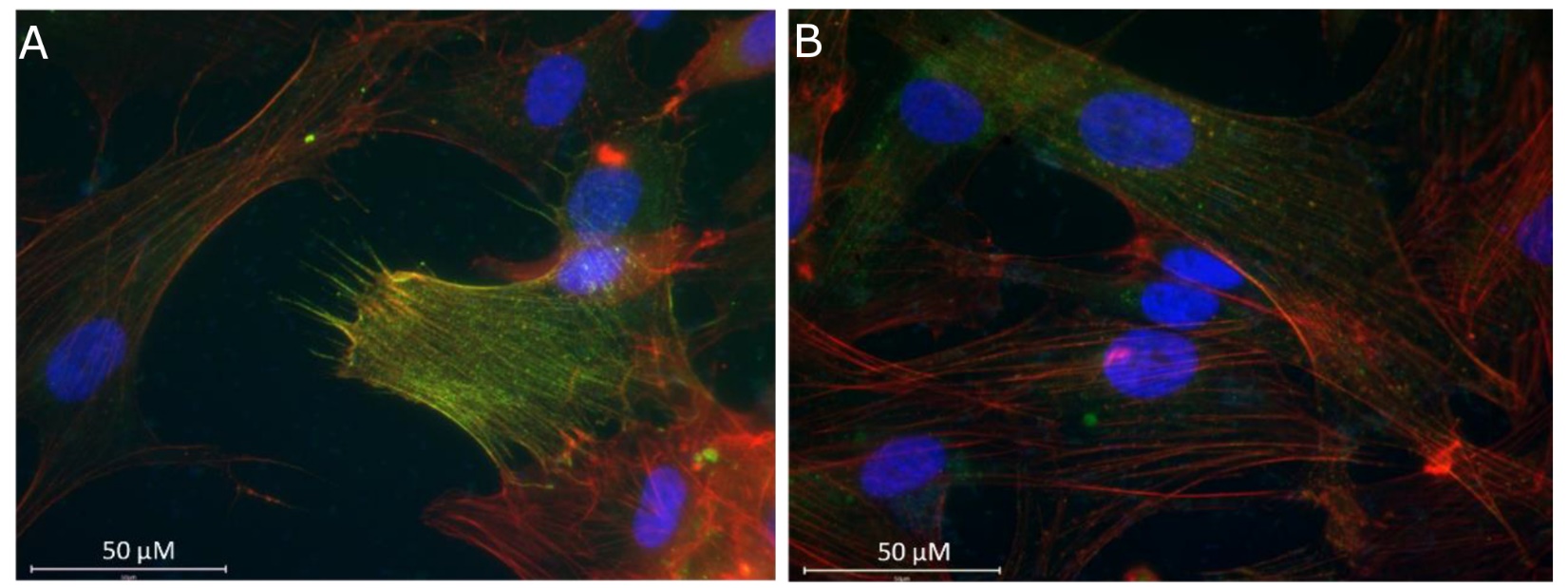


Table: Immunofluorescence antibodies

| **Antibody** | **Fluorochrome** | **Clone** | **Dilution** | **Manufacturer** |
| --- | --- | --- | --- | --- |
| α-SMA |  | D4K9N | 1:200 | CellSignaling |
| Goat anti-rabbit secondary antibody | AF488 |  | 1:1000 | ThermoFisher |
| Phalloïdine | DyLight™ 554 |  | 1:200 | Cell Signaling |
